# Yersinia pestis Δ*ail* Mutants Are Not Susceptible to Human Complement Bactericidal Activity in the Flea

**DOI:** 10.1128/aem.01244-22

**Published:** 2023-02-06

**Authors:** Anna M. Kolodziejek, Scott W. Bearden, Sarah Maes, John M. Montenieri, Kenneth L. Gage, Carolyn J. Hovde, Scott A. Minnich

**Affiliations:** a Department of Animal, Veterinary and Food Science, University of Idaho, Moscow, Idaho, USA; b Division of Vector-Borne Diseases, Centers for Disease Control and Prevention, Fort Collins, Colorado, USA; Unité Mixte de Recherche Processus Infectieux en Milieu Insulaire Tropical (UMR PIMIT). Université de La Réunion, INSERM 1187, CNRS 9192, IRD 249

**Keywords:** *Yersinia pestis*, plague, flea, Ail, LPS, serum resistance, complement, transmission

## Abstract

Ail confers serum resistance in humans and is a critical virulence factor of Y. pestis, the causative agent of plague. Here, the contribution of Ail for Y. pestis survival in the flea vector was examined. Rat or human but not mouse sera were bactericidal against a Y. pestis Δ*ail* mutant at 28°C *in vitro*. Complement components deposited rapidly on the Y. pestis surface as measured by immunofluorescent microscopy. Ail reduced the amount of active C3b on the Y. pestis surface. Human sera retained bactericidal activity against a Y. pestis Δ*ail* mutant in the presence of mouse sera. However, in the flea vector, the serum protective properties of Ail were not required. Flea colonization studies using murine sera and Y. pestis KIM6^+^ wild type, a Δ*ail* mutant, and the Δ*ail/ail^+^* control showed no differences in bacterial prevalence or numbers during the early stage of flea colonization. Similarly, flea studies with human blood showed Ail was not required for serum resistance. Finally, a variant of Ail (Ail^F100V E108_S109insS^) from a human serum-sensitive Y. pestis subsp. microtus bv. Caucasica 1146 conferred resistance to human complement when expressed in the Y. pestis KIM6^+^ Δ*ail* mutant. This indicated that Ail activity was somehow blocked, most likely by lipooligosaccharide, in this serum sensitive strain.

**IMPORTANCE** This work contributes to our understanding of how highly virulent Y. pestis evolved from its innocuous enteric predecessor. Among identified virulence factors is the attachment invasion locus protein, Ail, that is required to protect Y. pestis from serum complement in all mammals tested except mice. Murine sera is not bactericidal. In this study, we asked, is bactericidal sera from humans active in Y. pestis colonized fleas? We found it was not. The importance of this observation is that it identifies a protective niche for the growth of serum sensitive and nonsensitive Y. pestis strains.

## INTRODUCTION

Ancient DNA surveys from Neolithic and Bronze Age human remains unexpectedly contain full genomic sequences of Yersinia pestis, the Gram-negative bacterium that causes plague (1–5). These sequences compared against genomes of modern *Yersinia* have provided what is perhaps the most complete evolutionary trajectory of a zoonotic human pathogen with resolution to the nucleotide. Y. pestis diverged recently (<20,000 years ago) from an enteric predecessor, Y. pseudotuberculosis as enzootic strains belonging to Pestoides clusters (6). Their appearance was followed by a binary split around 6,500 years ago and produced strains more frequently associated with human disease (1). Interestingly, nonclassical Y. pestis strains (Pestoides clusters, including Microtus isolates) are distinct from the classical virulent lineages in that they usually do not cause severe, deadly, or transmissible human infections (7–9). Studies of the evolutionarily older strains indicate how the mildly virulent animal pathogen likely diverged into a highly virulent human pathogen capable of causing catastrophic pandemics.

Plague cycles between a mammalian host and a flea vector (10–15). The disease affects a wide range of mammals, including the felid family, rabbits, dogs, and primates; however, the primary mammalian reservoir are rodents and shrews (16–18). Vector-borne transmission to mammalian species is determined by flea feeding preferences. When the primary host population is decimated by the disease, the flea will feed on other mammalian species that can become a “bridge vector” to humans (16, 19, 20). Y. pestis can infect people by a flea bite (bubonic plague), by direct deposition of the bacteria into blood through contact with body fluids or tissue of an infected mammal (septicemic plague), or by inhalation of aerosolized bacteria (pneumonic plague). Septicemic and pneumonic plague are the deadliest forms of the disease, but bubonic plague is the most common (21).

Flea-borne plague transmission to mammals is possible throughout the early and late stages of insect infection. Early-phase flea infection, within a week after a contaminated blood meal, has the least efficient transmission rates, yet is still significant for plague maintenance (22–24). The hallmark of late-phase flea infection is biofilm formation on the proventriculus, a valve between the esophagus and the midgut that prevents meal backflow (11, 25). The thick biofilm restricts entrance of the blood meal to the midgut and is referred to as flea blockage. The starving flea goes into a feeding frenzy of continual biting, resulting in increased plague transmission (26, 27). Biofilm-contaminated blood is regurgitated into the flea esophagus and onto the mammalian skin bite. Thus, the late-phase infection is highly efficient in Y. pestis transmission. The bacterial genes for flea colonization and biofilm formation are regulated by temperature-dependent transcriptional and posttranscriptional factors (28–30).

Mammalian blood complement is part of the innate immune response recognizing and responding rapidly to pathogens (31). Complement activation generates a series of effector proteins that lead to formation of a membrane attack complex (MAC) that forms membrane-penetrating pores on the microbial surface. Complement can be activated by three biochemical pathways: the classical, the alternative, and the lectin. Each induces C3-convertases, enzymes that cleave the C3 component into anaphylatoxin C3a and opsonin C3b. This catalytic step in the complement cascade amplifies the reaction to decorate the microbe’s surface with the C3b component upon which the MAC assembles (31).

Mammalian blood complement exerts strong selective pressure on microorganisms to evade its lethal effects. The resulting microbial adaptations are referred to as bacterial serum resistance. Complement neutralization is a key virulence attribute of blood-borne pathogens. Maintenance of transmission cycles between preferred and incidental vector hosts requires serum resistance in both (7). The definitive suite of Y. pestis factors necessary for virulence in humans is still under investigation (10, 32); however, resistance to human complement is an absolute requirement. It is hypothesized that acquisition of serum resistance to human complement was pivotal for Y. pestis to become a human pathogen (7). For the related enteric pathogens, Y. pseudotuberculosis and Y. enterocolitica, the outer membrane proteins YadA and Ail, in concert with the O-antigen of lipopolysaccharide (LPS) are serum resistance factors. Interestingly, genes coding for Y. pestis YadA and O-antigen have been lost by mutation, leaving Ail as its single serum resistance factor (33, 34). Previously, we established that Y. pestis Ail confers resistance to human and rat sera at mammalian host temperature; a Y. pestis Δ*ail* mutant was sensitive to sera from these species and was killed within 1 h of exposure (35, 36). The result is confirmed by another study which further expanded the list to sera of other species: goat, sheep, rabbit, and guinea pig (37). In contrast, mouse serum is not bactericidal to the Δ*ail* mutant and no difference in survival is found between the Y. pestis KIM6^+^ wild type, the Δ*ail* mutant, or the Δ*ail/ail^+^* control at 37°C (36–38). Importantly, species-specific bactericidal action of complement against the Δ*ail* mutant at lower extrahost temperature has not been established. Testing various sera in the flea will lead to better understanding of Y. pestis colonization and survival in its vector.

Some of the nonclassical Y. pestis strains also show species variability to serum. For example, Y. pestis subsp. *microtus* bv. Caucasica, found in the East Caucasian mountains, is a representative of one of the most ancient lineages, 0.PE2 (15). It is sensitive to human serum, but not mouse serum (7, 39). This phenotype reflects the pattern of host morbidity and mortality of this strain. In laboratory conditions it is virulent in mice, but either avirulent or of low virulence in guinea pigs. Furthermore, in its natural reservoir it was found to circulate in populations of voles with extremely rare, nonlethal infections when transmitted to humans (7, 9). Host innate immunity mechanisms, particularly the bactericidal potential of complement, was proposed to determine species-dependent susceptibility to plague infection (7, 39). The dynamics of plague vector maintenance in environments sharing variable serum resistant hosts, as well as the contribution of Ail, is understudied.

Previously, it was determined that Ail is not required for the colonization of fleas feeding on murine blood in which complement is not bactericidal (37). This study evaluated whether Ail was required to protect Y. pestis in *Oropsylla montana* fleas fed human blood meals containing bactericidal complement. The role of Ail in C3b deposition on the Y. pestis surface at mammalian host and flea vector temperatures was assessed. The significance of Ail amino acid variations in serum sensitive Y. pestis subsp. microtus bv. Caucasica 1146 was evaluated. Flea infections with Y. pestis KIM6^+^ wild type, the Δ*ail* mutant, or the *ail/ail^+^* control and feedings with mouse or human blood were compared. Heterogenous sensitivity to serum of some nonclassical Y. pestis strains is discussed.

## RESULTS

### The complement cascade was activated rapidly, and components deposited on the Y. pestis surface at both mammalian and at 28°C.

**FIG 1:**
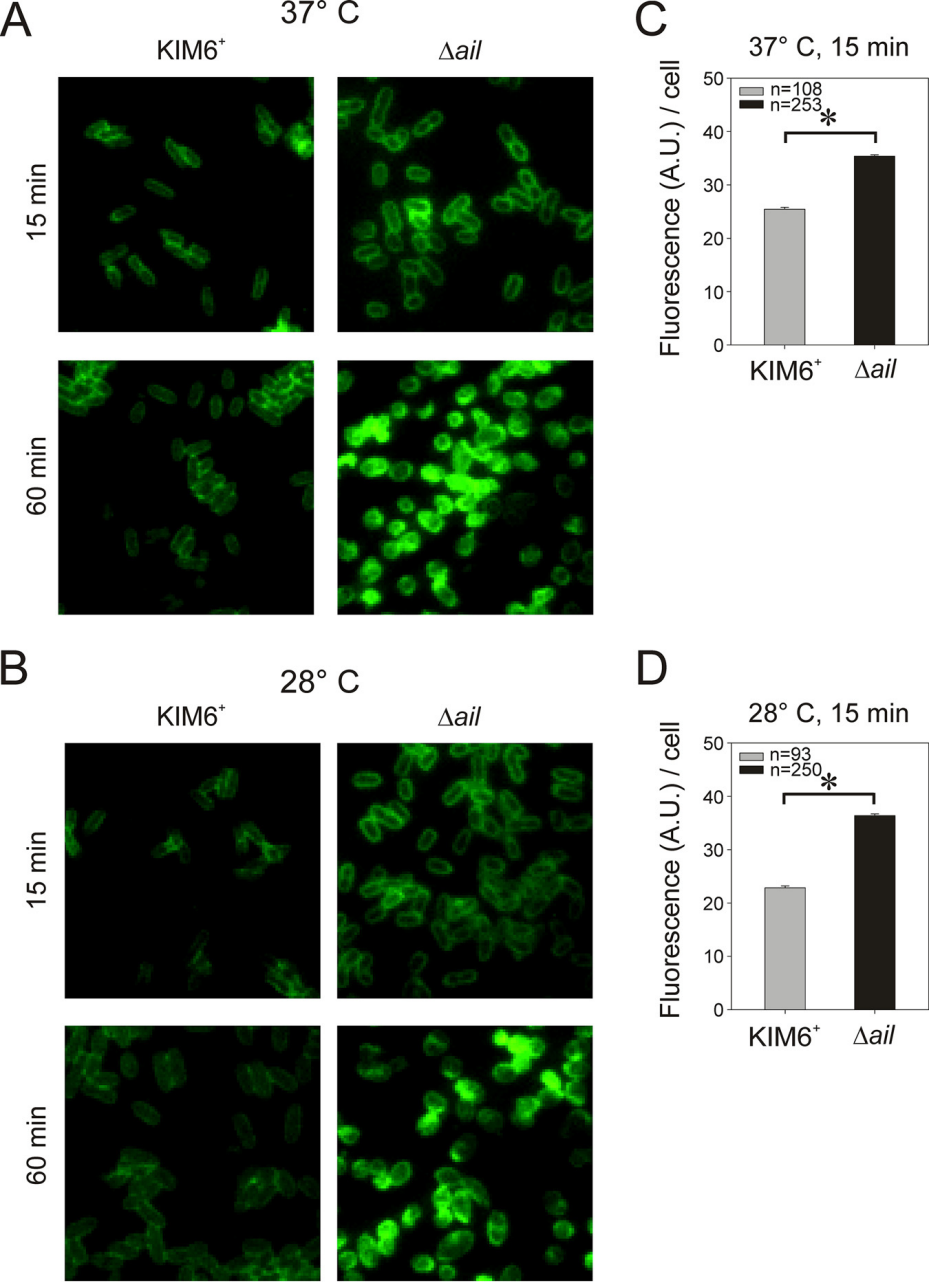
The complement cascade was activated rapidly, and components deposited on the Y. pestis surface at both mammalian and 28°C temperatures. Y. pestis wild-type KIM6*^+^* and the Δ*ail* mutant were grown with aeration to midexponential phase at 28°C in Lysogeny broth (LB). Cells were collected, washed with PBS, and incubated in 50% normal human serum (NHS) or heat-inactivated serum (HIS) for 15 or 60 min at 37°C (A) or 28°C (B). Immunofluorescence (green) shows C3b on bacterial surfaces as detected with polyclonal goat anti-C3 and rabbit FITC-conjugated anti-goat IgG. Observations were with a Nikon Eclipse E1000 fluorescence microscope at 1000×; representative cells are shown. MicrobeJ software was used to quantify the C3b component deposition on wild-type KIM6^+^ or Δ*ail* cells after 15 min incubation with NHS at 37°C (C) or 28°C (D). Background-subtracted FITC fluorescence arbitrary units (A.U.)/cell were determined. Aggregated bacteria were excluded from the analysis and only individual bacteria were used for quantification. Results are means ± SE, asterisk (*) indicates statistical difference (Mann-Whitney Rank Sum test, *P* < 0.001).

Previous results from serum resistance assays show human serum kills Δ*ail* mutant at 37°C within 1 h by activation of the complement cascade (35). To assess complement killing at a lower (extrahost) temperature, KIM6^+^ wild type and the Δ*ail* mutant were incubated in 50% normal human serum (NHS) or heat-inactivated serum (HIS) at 28°C or 37°C, and immunostained with polyclonal anti-C3 serum. Immunofluorescent imaging detected human C3b on the surfaces of KIM6^+^ wild type and the Δ*ail* mutant 15 min after exposure to NHS at both mammalian host and 28°C temperatures (Fig. 1). KIM6^+^ wild type and the Δ*ail* mutant cells decorated with C3b retained their morphology at this time point. Only the Δ*ail* mutant showed signs of lysis that included swelling and rounding after 60 min of incubation at either temperature. This phenotype was not observed with the KIM6^+^ wild type. C3b in HIS serum controls was not detected. In addition, single-cell quantification of C3b using immunofluorescence images showed C3b deposition on the Y. pestis surfaces was Ail-dependent. Significantly larger amounts of C3b (Mann-Whitney Rank Sum test, *P* < 0.001) were found on the Δ*ail* mutant cells compared to the KIM6^+^ wild-type cells at either 28°C (Fig. 1C) or 37°C (Fig. 1D).

These data indicated that the human complement cascade was activated rapidly after contact with Y. pestis at 28°C and the Δ*ail* mutant showed signs of lysis within 1 h. In addition, lysis correlated with increased C3b on cell surfaces.

### Rat or human but not mouse sera were bactericidal against the Y. pestis Δ*ail* mutant at 28°C.

**FIG 2:**
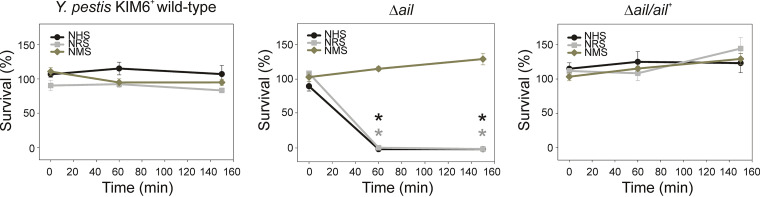
Rat or human but not mouse sera were bactericidal against the Y. pestis Δ*ail* mutant at 28°C. Y. pestis wild-type KIM6*^+^*, the Δ*ail* mutant, and Δ*ail*/*ail^+^* were grown with aeration to midexponential phase at 28°C in Lysogeny broth (LB). Cells were centrifuged, washed with PBS, and incubated in 50% normal human serum (NHS), normal rat serum (NRS), normal mouse serum (NMS), or the respective heat-inactivated sera (HIS) for 0, 60, and 150 min at 28°C. Bacteria were enumerated by plate counts and CFU after each HIS incubation were assigned as 100% survival. Results are means ± SEM (*n* = 4) from experiments performed twice in duplicate. Asterisk (*) indicates statistical differences between mouse compared to human or rat sera (repeated-measures ANOVA, *P* < 0.001).

To determine if sera other than human was bactericidal against the Δ*ail* mutant at lower temperature, rat and mouse sera were tested. KIM6*^+^* wild type, the Δ*ail* mutant, or the Δ*ail*/*ail*^+^ complement were incubated at 28°C in 50% active human, rat, or mouse serum for 0, 60, or 150 min. Bacteria were enumerated by plate counts and the number after incubation with HIS was designated 100% survival. Data indicated that 28°C supported adequate thermal conditions for efficient activation of rat and human complement and killing of the Δ*ail* mutant (Fig. 2). Parental and complemented strains retained full viability at all time points. Rat and human sera were similarly bactericidal for the Δ*ail* mutant. Mouse serum was not lethal as there was no difference in survival among the Δ*ail* mutant, the KIM6*^+^* wild type, or the Δ*ail*/*ail*^+^ complement (Fig. 2).

These observations indicated that the bactericidal effects of human and rat complement against the Δ*ail* mutant occur at the lower flea vector temperature.

### Human sera retained bactericidal activity against the Y. pestis Δ*ail* mutant in the presence of mouse sera.

**FIG 3:**
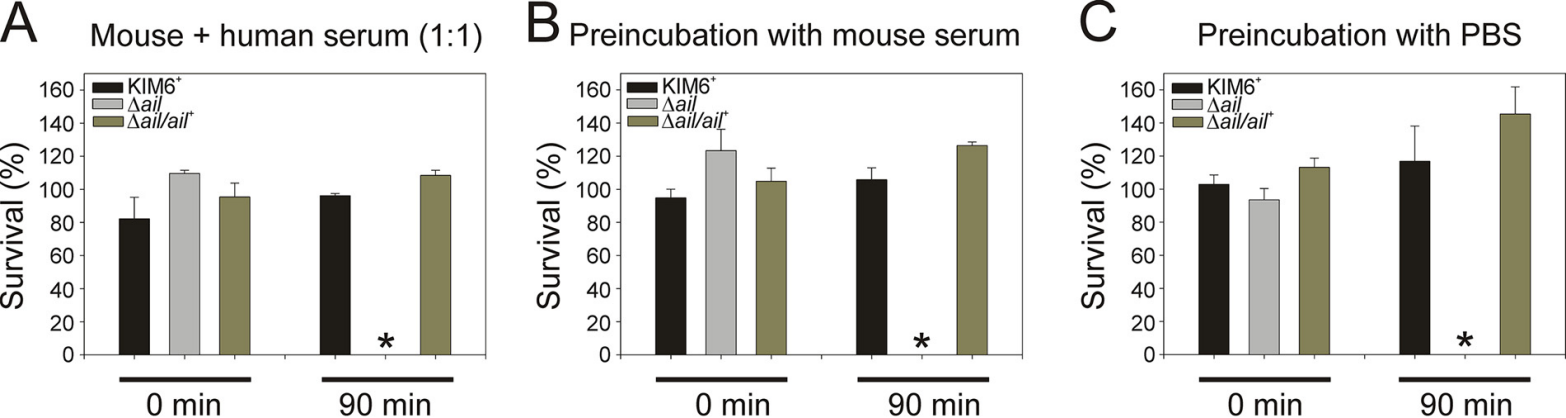
Human sera retained bactericidal activity against the Y. pestis Δ*ail* mutant in the presence of mouse sera. Y. pestis wild-type KIM6*^+^*, the Δ*ail* mutant, and Δ*ail*/*ail^+^* were grown with aeration to midexponential phase at 28°C in Lysogeny broth (LB). Cells were centrifuged, washed with PBS, and mixed (1:1) with: 50% normal mouse sera (NMS) and normal human sera (NHS) or 50% of the respective heat-inactivated sera (HIS) (A), 50% NMS (B), or PBS (C). Cells were incubated for 0 or 90 min followed by plate count enumeration (A). Cells were preincubated for 60 min and equal volumes of NHS or HIS were added (B, C). Samples were further incubated for 0 or 90 min followed by plate count enumeration. All incubations were performed at 28°C. CFU after each HIS incubation were assigned as 100% survival. Results are means ± SEM (*n* = 4) from experiments performed twice in duplicate. Asterisk (*) indicates statistical differences (ANOVA, *P* < 0.001) at 90 min between the Δ*ail* mutant and the indicated strain.

Because mouse sera failed to kill the Δ*ail* mutant (Fig. 2) we asked if mouse sera could inhibit the bactericidal effects of other sera. KIM6*^+^* wild type, the Δ*ail* mutant, and the Δ*ail*/*ail*^+^ complement were incubated at 28°C in a combined ratio of 1:1 mouse and human sera. After 90 min, no Δ*ail* mutant cells survived, similar to incubation with human serum only (Fig. 3A). In addition, preexposure of bacteria for 1 h to mouse sera did not suppress the bactericidal effect of human sera (Fig. 3B) compared to controls (Fig. 3C). All reactions were performed at 28°C. This showed that mouse sera did not suppress the bactericidal properties of human serum. The results also suggested that human serum would remain bactericidal against the Δ*ail* mutant in the flea vector even if the flea had previously fed on a host with ineffective complement.

### Loss of Ail does not affect prevalence or bacterial number in the early stage of flea colonization.

**FIG 4:**
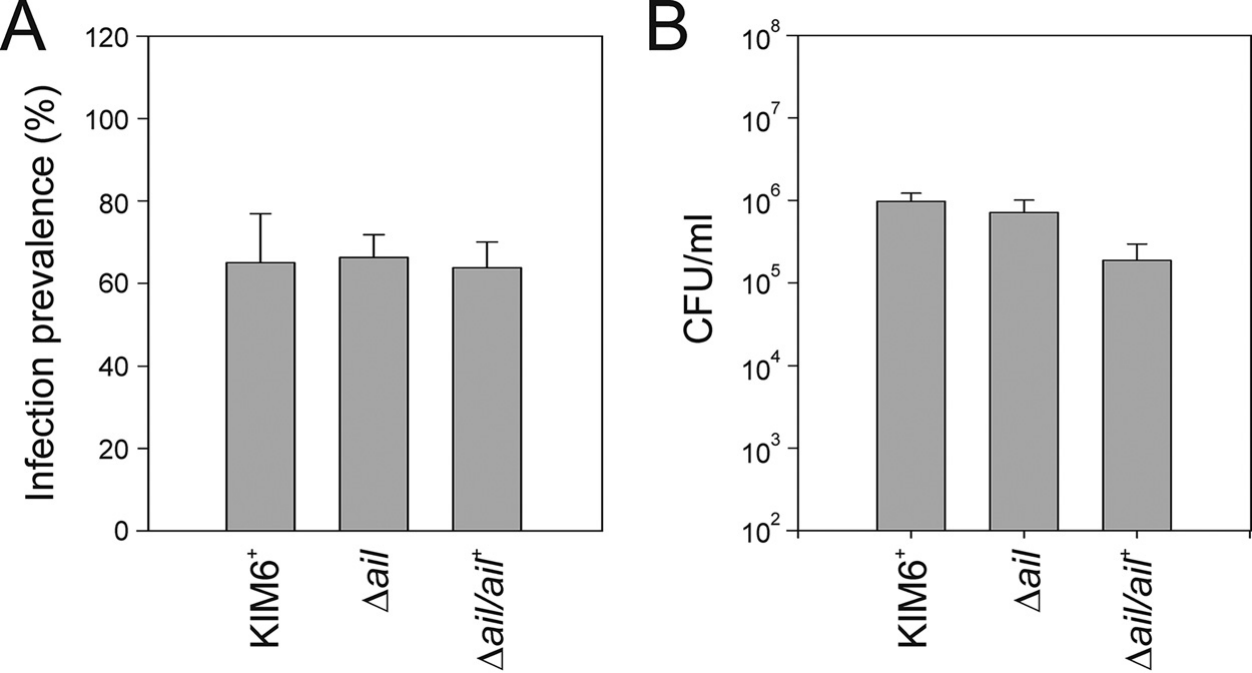
Loss of Ail does not affect infection prevalence or bacterial number in the early stage of flea colonization. Y. pestis wild-type KIM6*^+^*, the Δ*ail* mutant, or Δ*ail*/*ail^+^* were suspended in defibrinated Swiss Webster mouse whole blood and fed to fleas. At 72 h fleas were collected, individually homogenized in 10% glycerol brain heart infusion broth and Y. pestis enumerated by plate count. The percent prevalence of infected fleas (A) and average CFU/mL/flea (B) were assessed 72 h postinfection. Results are the means ± SEM (*n* = 50 fleas/strain) from experiments performed four times on separate days.

Bartra et al. show that in late stages of mouse-flea infections, Ail-dependent autoaggregation of Y. pestis is not required for late stages of biofilm formation or flea proventriculus blockage at 4-weeks postinfection (37). Y. pestis is transmissible from fleas to mammalian hosts during the early stages of infection when flea blockage has not yet occurred. Formation of bacterial aggregates in the flea gut is characteristic for this stage. To determine if Ail-mediated Y. pestis autoaggregation is important for early-stage flea colonization, infections with Y. pestis KIM6*^+^* wild type, the Δ*ail* mutant, or the Δ*ail*/*ail*^+^ control suspended in mouse whole blood were done and evaluated at 72 h (Fig. 4). A total of 50 fleas/strain was assayed for infection prevalence and bacterial numbers. Infection prevalence was not different and averaged 65%, 66.25%, 63.75%, for Y. pestis KIM6*^+^* wild type, the Δ*ail* mutant, or the Δ*ail*/*ail*^+^ control, respectively (Fig. 4A). Similarly, average bacterial number/flea was not different between the Δ*ail* mutant and the Y. pestis KIM6*^+^* wild type or the Δ*ail*/*ail*^+^ control (*t* test, *P* = 0.927 and *P* = 0.775, respectively; Fig. 4B).

These observations indicated that Ail and its autoaggregation phenotype were not required for the early stage of Y. pestis flea colonization.

### Loss of Ail does not confer susceptibility to human complement bactericidal activity in fleas colonized with Y. pestis.

**FIG 5:**
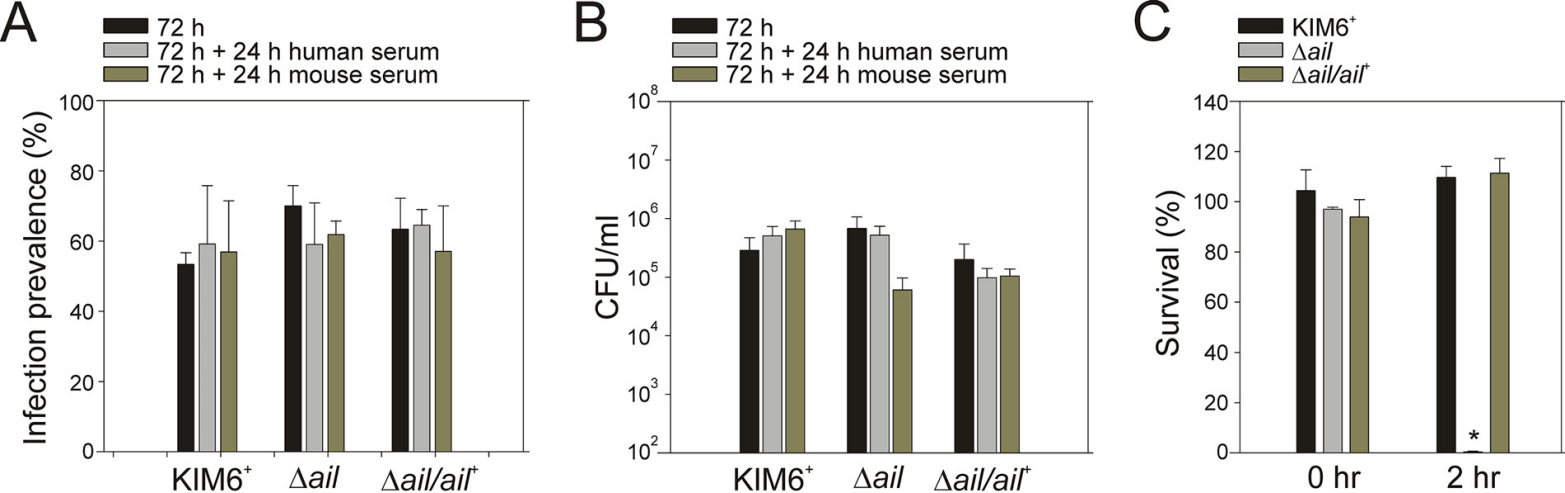
Loss of Ail does not confer susceptibility to human complement bactericidal activity in fleas colonized with Y. pestis. Y. pestis wild-type KIM6*^+^*, the Δ*ail* mutant, or Δ*ail*/*ail^+^* were suspended in defibrinated Swiss Webster mouse whole blood and fed to fleas. Maintenance feeds with sterile human whole blood (gray) or mouse whole blood (green) were performed 72 h later. Fleas were collected at 72 h (before the maintenance feed; black) and at 96 h (24 h after the maintenance feed), individually homogenized in 10% glycerol brain heart infusion broth and Y. pestis enumerated by plate count. The percent prevalence of infected fleas (A) and the average CFU/mL/flea (B) were assessed. Results are means ± SEM (*n* ≥ 30/strain/condition) from experiments performed thrice on separate days. The human blood used in these feeding trials was tested for complement activity by standard protocol using each Y. pestis strain. Complement activity is evident by serum sensitivity of the Δ*ail* mutant (C).

Because human complement was bactericidal against the Δ*ail* mutant at 28°C *in vitro* (Fig. 1B, Fig. 2) we tested if loss of Ail had the same effect in the flea gut after a human blood meal. Fleas were infected with Y. pestis KIM6*^+^* wild type, the Δ*ail* mutant, or Δ*ail*/*ail*^+^ control with mouse whole blood (infectious feed). After 72 h fleas were fed sterile mouse or human whole blood (maintenance feed) and housed for another 24 h. Infection prevalence and bacteria numbers in fleas were assessed (Fig. 5A, 5B). Infection prevalence and bacterial numbers were not different for the Δ*ail* mutant compared to the other strains (*P* = 0.935 and *P* = 0.293, respectively, ANOVA). In contrast to *in vitro* studies where human complement was bactericidal against the Δ*ail* mutant (Fig. 5C) even when mixed with mouse serum *in vitro* (Fig. 3), these results showed that exposure of the Δ*ail* mutant to human blood in the flea gut did not reduce bacterial numbers.

### A variant of Ail (Ail^F100V E108_S109insS^) from a human serum-sensitive Y. pestis subsp. *microtus* bv. Caucasica 1146 conferred resistance to human complement when expressed in the Y. pestis KIM6^+^ Δ*ail* mutant.

**FIG 6:**
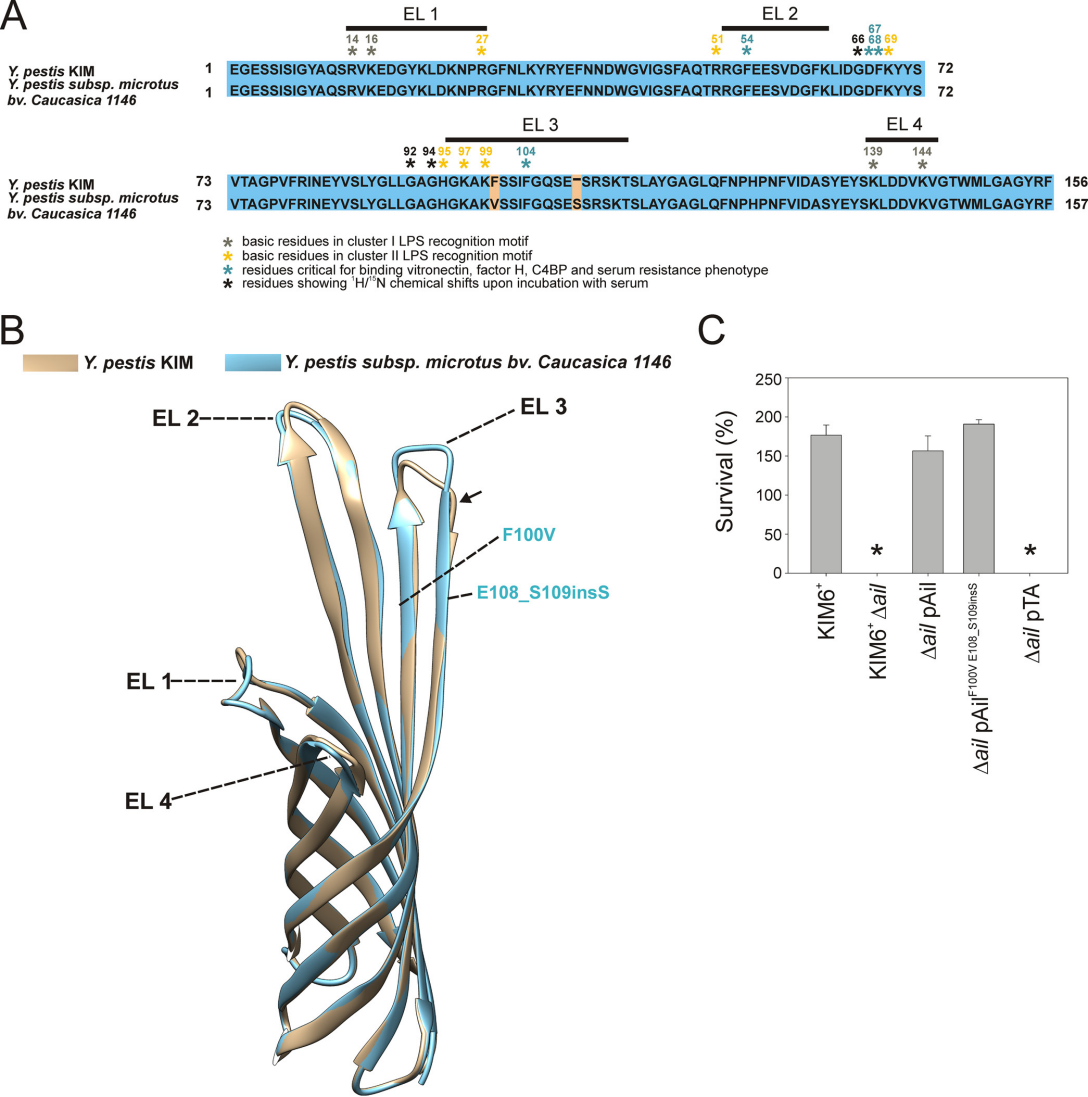
A variant of Ail (Ail^F100V E108_S109insS^) from a human serum-sensitive Y. pestis subsp. microtus bv. Caucasica 1146 conferred resistance to human complement when expressed in Y. pestis KIM6^+^. Sequence alignment of Ail from Y. pestis KIM6+ and Y. pestis subsp. microtus bv. Caucasica 1146 (A). Residue numbers correspond to the mature Ail without the signal sequence. Amino acids known to be involved in LPS interactions (43) and serum resistance phenotypes (41–43) are indicated by asterisks. (B) Y. pestis KIM6^+^ and Y. pestis subsp. microtus bv. Caucasica 1146 Ail structural models were generated with RoseTTAFold and superimposed with UCSF Chimera plug-in, MatchMaker. Arrow indicates the extended β-sheet motif in Ail from Y. pestis subsp. microtus bv. Caucasica 1146. Extracellular loops (EL 1, EL 2, EL 3, and EL 4) and positions of variable amino acids are shown. Y. pestis wild-type KIM6*^+^*, the Δ*ail* mutant, Δ*ail* pAil, Δ*ail* pAil^F100V E108_S109insS^, and Δ*ail* pTA (vector control) were grown with aeration to midexponential phase at 28°C in Lysogeny broth (LB). Cells were centrifuged, washed with PBS, and incubated in 50% normal human serum (NHS) or heat-inactivated human serum (HIS) for 90 min at 28°C. Bacteria were enumerated by plate count and CFU after each HIS incubation were assigned as 100% survival (C). Results are means ± SEM (*n* = 4) from experiments performed twice in duplicate. Asterisk (*) indicates statistical difference from wild-type KIM6*^+^* (repeated-measures ANOVA, *P* < 0.001).

The amino acid sequence of Y. pestis Ail is highly conserved among all strains and has high sequence homology (91 to 100%) with Ail from Y. pseudotuberculosis strains (National Health Institute, NIH, GenBank, ClustalW analysis). BLAST analyses showed Ail sequences from the main, human-infectious Y. pestis biovars (Mediaevalis, Orientalis, Antiqua) either retain 100% homology or had one amino acid substitution (S102P or F100V), while sequences of nonclassical biovar Pestoides had up to 3% variability (NIH GenBank, ClustalW analysis). Ail from the human serum-sensitive Y. pestis subsp. microtus bv. Caucasica 1146 strain has the F100V substitution and an additional S insertion at position 108 (Fig. 6A). These changes are in the extracellular loop 3 (EL 3) that is involved in binding vitronectin, factor H, C4BP, as well as maintaining the Y. pestis serum resistance phenotype (Fig. 6A) and (40–44). Superimposed Ail variant structural models generated with RoseTTFold showed extension of the β-sheet motif and, as a result, shortening of the EL 3 loop in Ail^F100V E108_S109insS^ (Fig. 6B). In addition, F100 is in close proximity to a group of basic residues (cluster II) important for Ail interactions with LPS (Fig. 6A) and (43). To determine if these amino acid changes affected Ail-mediated human complement sensitivity, the variant Ail^F100V E108_S109insS^ was cloned in the pTA vector, expressed in the KIM6*^+^* Δ*ail* mutant, and tested for serum resistance. KIM6*^+^* wild type, the Δ*ail* mutant, Δ*ail* pAil, Δ*ail* pAil^F100V E108_S109insS^, and Δ*ail* pTA (vector control) were incubated in 50% NHS for 90 min at 28°C. Expression of Ail^F100V E108_S109insS^ fully restored human serum resistance of the Δ*ail* mutant (Fig. 6C). There was no difference in survival among the KIM6*^+^* wild type, the Δ*ail* pAil, and the Δ*ail* pAil^F100V E108_S109insS^ strains (*P* = 0.34, ANOVA; Fig. 6C). Human serum was only bactericidal for the Δ*ail* mutant and the Δ*ail* pTA vector control.

This result showed that the serum sensitivity of Y. pestis subsp. microtus bv. Caucasica 1146 was not due to amino acid differences in Ail because this protein was functional and conferred serum resistance when expressed in the KIM genetic background.

## DISCUSSION

Ail is a critical virulence factor because it protects Y. pestis from serum complement (35, 36, 45). Here, we further evaluated the contribution of Ail to serum resistance at 28°C, the optimum temperature for Y. pestis growth. First, we showed that the Δ*ail* mutant was sensitive to human serum at 28°C *in vitro*. Second, because mouse serum is not lethal to the Δ*ail* mutant, we showed mixing mouse with lethal human sera provided no protection. Based on these *in vitro* observations, we then asked if the Δ*ail* mutant, established in fleas feeding on mouse blood, could survive when these fleas were switched to a human blood meal. Surprisingly, Δ*ail* mutant levels in fleas were not affected compared to wild-type infected fleas, or control fleas maintained on mouse blood. This *in vivo* result implied that fleas rapidly inactivate human complement upon blood ingestion or the Y. pestis growing in fleas are protected in some manner from complement activity. This inactivation or protection from complement could, therefore, maintain a murine reservoir of Y. pestis strains with heterogenous complement sensitivity, even if fleas changed mammalian hosts. Finally, several avirulent or low virulent natural isolates of Y. pestis are serum-sensitive and display altered Ail amino acid sequences. By cloning one of these *ail* variants from serum-sensitive Y. pestis subsp *microtus* bv. Caucasia and expressing it in the serum-sensitive *Y. pesits* KIM6^+^ Δ*ail* mutant, we showed serum resistance was restored by the variant Ail protein.

Previous studies show that Ail is not required for flea blockage (37). These experiments were conducted in mice and consistent with the lack of serum complement lethality of murine blood. Our studies further contribute to these observations. Pre- and coincubation of human serum with mouse serum, modeling consecutive feedings on different hosts *in vitro*, showed that mouse serum does not have any inhibitory properties on human complement activity against Δ*ail* mutant lysis. In contrast, our *in vivo* flea studies showed both colonization and prevalence of Y. pestis KIM6^+^ wild type and the Δ*ail* mutant did not change when fleas, colonized with infected murine blood, took a human blood meal. This suggests the inability to clear the Δ*ail* mutant by human serum in the flea is due to the rapid inactivation of complement. Fleas take about 6 h to hemolyze cells and digest a blood meal (27, 46). Our previous results show that bactericidal activity of serum shows an ~10-fold reduction of Δ*ail* mutant cells *in vitro* within 30 min (35). Data from fluorescence microscopy imaging presented here confirmed that activation of human complement is rapid upon contact with Y. pestis cells and much shorter than the time needed for blood meal digestion in fleas. Hence, the inability of human complement to clear bacteria from the flea’s digestive tract suggests the presence of innate mechanisms neutralizing complement components. Anti-complement activity is detected in saliva and the digestive tracts in several, phylogenetically distinct hematophagous arthropods, including ticks, sand flies, mosquitoes, and triatomines (47–51). Several protein candidates for this inhibitory activity have been identified (52–54). It is suggested that the primary role of these inhibitors is to protect arthropod cells lining the digestive tract from the detrimental action of host complement present in a blood meal (49, 50). This inhibition of complement activity in fleas could likewise benefit the pathogens they transmit, either with their colonization or transmission during saliva injection into the bite site (53). To our knowledge, such activity in flea salivary extracts or the midgut has not been reported; however, based on the physiology of other hematophagous insects and our study, it seems likely that these mechanisms are also present in Siphonaptera. Thus, Y. pestis and other vector-borne pathogens, appear to capitalize on arthropod complement inactivation for vector colonization and subsequent transmission to mammals.

Y. pestis biofilm formation in the proventriculus and midgut of fleas is also a necessary adaptation for plague transmission (25, 26, 55). Bacterial autoaggregation is a phenotype associated with biofilm formation (56). Y. pestis aggregation is observed through the whole infection process in the flea, beginning with formation of aggregates during initial attachment to the proventriculus surface (27). In contrast to wild-type Y. pestis, the Δ*ail* mutant does not form a pellicle or autoaggregate in static media *in vitro* (35–37, 44). Ail also confers this phenotype when expressed in E. coli, but only with rough LPS. Autoaggregation is blocked in E. coli-expressing Ail with full-length O-antigen polysaccharide (36). Loss of Y. pestis autoaggregation in the Δ*ail* mutant suggested that this protein could also contribute to biofilm formation in fleas and plague transmission in concert with other biofilm inducing genes. However, studies by Bartra et al. show that deletion of *ail* does not reduce proventricular blockage in *X. cheopsis* fleas (37). In their study, flea populations were infected with mouse blood containing the Δ*ail* mutant or its parental strain. These populations were maintained on mouse blood feedings for 4 weeks and did not show any differences in flea-blockage or bacterial loads at the end of this trial (37). Our data confirm and expand these findings; the Δ*ail* mutant showed no difference compared to the parental wild type during the initial stages of vector colonization and after switching to feeding on ‘restrictive’ human blood. The recent work of Dewitte et al. (57) showing casts composed of large numbers of Y. pestis in the lumen of the proventriculus may also play a role in protection against serum complement.

Ail protein sequence variations and human serum-sensitivity displayed by some Y. pestis strains warrants investigation. We showed here the Ail variant sequence present in the human serum-sensitive strain Y. pestis subsp. *microtus* bv Caucasica (Ail^F100V E108_S109insS^), confers serum-resistance when expressed in the Y. pestis KIM6 *Δail* mutant. Interestingly, expression of Y. pestis KIM6 Ail in the serum sensitive Pestoides strain, Y. pestis 1680, did not confer serum resistance (preliminary result, data not shown). Taken together, these two observations indicate Ail-dependent serum resistance is regulated by another Y. pestis subspecies-specific factor. Previously, we observed that LOS structure contributes to Ail function, including Ail-dependent serum resistance, autoaggregation, and the adhesion and invasion capability of Y. pestis (36). Recent NMR data provide a structural explanation for this phenomenon, revealing specific Ail-LOS interaction sites and their importance for Ail activity (43, 58). Consistent with this thinking, it has been shown that atypical serum-sensitive Y. pestis strains have structurally different LOS (59–61). It seems plausible that species-dependent serum-sensitivity observed in some atypical strains is determined by LOS contributions to Ail structure and/or accessibility on the cell surface. It is well established that O-antigen loss in Y. pseudotuberculosis LPS was a critical step in the transition to the highly virulent Y. pestis (33, 62). These findings further support this idea.

Emergence of Y. pestis from enteropathogenic Y. pseudotuberculosis included gain of novel virulence factors as well as decisive genome reduction. Both changes ultimately led to i) development of very efficient mechanisms to subvert mammalian host immune responses and ii) acquisition of a bi-phasic life cycle shared between mammal and flea hosts (10–15). Serum-sensitive strains of Y. pestis, like the Microtus strain could be remnants of populations that never made this transition to serum resistance and have adapted to, and remain restricted to, hosts that lack lethal complement activity. The development of resistance to serum complement has been suggested as one of the key determinants of pathogenicity for plague (7).

Y. pestis has a reduced arsenal to combat the complement system due to genetic reduction. O-antigen and YadA, found to play a dominant role in the serum resistance of Y. pseudotuberculosis *and* Y. enterocolitica, are not present in Y. pestis. Ail interacts with the complement cascade regulator, C4b-binding protein (C4BP) (63). Ail binding to C4BP inhibits the C4b component and subsequently inactivates C4b2a C3 convertase which generates C3b. In addition, Ho et al. reported that Ail binds noncovalently purified C3b protein (63) and hypothesized that Ail-binding facilitates C3b cleavage and inactivation on the bacterial surface. These mechanisms could explain the lower level of deposition of C3 on the Y. pestis KIM6+ surface compared to the Δ*ail* mutant after incubation with human serum reported here.

Due to the recent introduction to the American continent, Y. pestis strains are mostly homogenous with exception of a few isolates in Brasil (64). In contrast, Asia is the most diverse region in terms of strains and lineages (7). Localities in Central Asia and the Caucasus maintains foci of ancient plague progenitor strains (6, 7, 65). Y. pestis phenotypic and genetic strain heterogeneity in the same, natural area of endemicity provides valuable insights into the dynamics of plague evolution (66). It includes the natural presence of nonclassical (Pestoides clusters, including human serum sensitive strains) and classical plague strains. Their patterned time and spatial coexistence provide opportunities for DNA exchange between strains, promoting their heterogeneity. It will be interesting to track LOS structural changes, evaluate their effect on Ail-mediated serum resistance, and establish its role for emergence of human virulent strains.

### Conclusion.

This study focused on the role of Ail in the flea vector. A Y. pestis *Δail* mutant was not affected by murine serum but was rapidly killed by human or rat sera *in vitro* at 28°C. Fleas were colonized with the Y. pestis *Δail* mutant while feeding on mouse blood. Surprisingly, Y. pestis *Δail* mutant numbers were not affected when fleas were subsequently provided human blood meals. This showed human complement was rapidly inactivated by fleas upon blood ingestion. Thus, Ail was not required for Y. pestis survival in the flea.

## MATERIALS AND METHODS

### Strains, plasmids, and media.

Strains and plasmids used in the study are listed in Table 1. Strains were cultured in Lysogeny low-salt broth (LB) (EMD Millipore, Burlington, MA) or brain heart infusion broth (BHI; Difco, Sparks, MD) at indicated temperatures with or without kanamycin (Kn), 50 μg mL^−^ or chloramphenicol (Cm), 30 μg mL^−^. Congo red agar (67) was used to confirm pigmentation phenotype of the Y. pestis KIM6*^+^* strains.

**TABLE 1:** Strains and plasmids used in the study

Strain or plasmid	Genotype and/or relevant characteristics	Reference or source
Strains		
Y. pestis		
KIM6*^+^* Nal^R^	*pgm*^+^ pCD1-pMT1^+^ pPCP^+^ Nal^R^	35
KIM6^+^Nal^R^ *Δail*::*npt* (previously *ΔompX*::*npt*)	*pgm*^+^ pCD1-pMT1^+^pPCP^+^ Δ*ail*; Nal^R^ Kn^R^	35
KIM6^+^Nal^R^ *Δail*	*pgm*^+^pCD1-pMT1^+^ pPCP^+^Δ*ail*; Nal^R^	71
KIM6^+^Nal^R^ *Δail*::*npt/ail*^+^ (previously *ΔompX*::*npt/ompX*^+^)	*pgm*^+^ pCD1-pMT1^+^pPCP^+^ *ail*^+^; Nal^R^ Kn^R^ Cm^R^	35
KIM6	*pgm-p*CD1-pMT1^+^ pPCP^+^	S. Straley, University of Kentucky
Pestoides F	*pgm*^+^ pCD1-pMT1^+^ pPCP-	Centers for Disease Control, Fort Collins, CO
Plasmids		
pCR2.1-TOPO	Amp^R^, Kn^R^	Invitrogen
pAil (previously pOmpX)	*ail* with its native promoter amplified from Y. pestis KIM6*^+^* Nal^R^ and cloned into pCR2.1-TOPO; Amp^R^, Kn^R^	36, 37
pnAil^F100V E108_S109insS^	*ail*_F126V E134_S135insS_ with its native promoter amplified from Y. pestis Pestoides F and cloned into pCR2.1-TOPO; Amp^R^, Kn^R^	This study
pTA	*ail*^−^ control plasmid derived from pAil by deletion of the fragment between EcoR*I* restriction enzyme-cut sites; Amp^R^, Kn^R^	36, 37

### Immunofluorescence microscopy.

Human C3 complement deposited on the Y. pestis surface were detected with primary goat polyclonal anti-C3 sera (SC-14612, Santa Cruz Biotechnology, Inc., Dallas, TX) and secondary rabbit FITC-conjugated anti-goat IgG (F2016, Sigma-Aldrich, St. Louis, MO), both diluted 1:500 in 1% bovine serum albumin in PBS. KIM6*^+^* wild type and the Δ*ail* mutant were incubated in LB broth, with or without Kn, at 28°C with aeration until midlogarithmic phase (OD_600_ = 0.8). Cells were collected (4,000 × *g*, 5 min, 4°C), washed twice in PBS, and mixed with 50% normal human serum (NHS) or human heat-inactivated serum (HIS) as described in the serum resistance assay. Cells were incubated for 15 or 60 min at 28°C or 37°C, pelleted by centrifugation (4,000 × *g*, 5 min, 4°C), washed twice, and resuspended in PBS. Bacteria were placed on poly-L-lysine-coated glass slides (Sigma-Aldrich, St. Louis, MO), air-dried at 24°C, and fixed with cold methanol (−20°C) for 5 min at RT. Samples were blocked with 1% bovine serum albumin (BSA) in PBS for 30 min, washed once, incubated with anti-C3 sera for 45 min at 37°C, washed thrice with PBS for 5 min, and stained with FITC-conjugated anti-goat IgG. After 45 min at 37°C, glass slides were washed thrice with PBS, once with deionized water, air-dried, covered with immersion oil (Sigma-Aldrich), and observed with a Nikon Eclipse E1000 fluorescence microscope (Tokyo, Japan) using a 100× objective. Images were taken with a Hamamatsu Orca digital camera (Hamamatsu, Japan) and Metamorph software (Molecular Devices; San Jose, CA).

### C3 complement quantification.

To quantify C3 on wild-type KIM6^+^ and Δ*ail* mutant cells, FITC fluorescent images were saved as 16-bit.tif files and analyzed with ImageJ plug-in MicrobeJ (68). To avoid bias from limited complement or anti-C3 antibody on aggregated bacteria, only individual cells were analyzed using the MicrobeJ settings at pixel units: area = 100 to 300, length = 15 to 30, width = 5 to 20, circularity = 0.10-max, curvature = 0-max, sinuosity = 1-max, angularity = 0 to 0.5, solidity = 0.8-max, and intensity = 0-max. Background-subtracted FITC fluorescence arbitrary units (A.U.)/cell were determined.

### Serum resistance assay.

Serum resistance assays were performed as described previously (35, 36). Briefly, HIS was prepared by incubation of fresh serum at 56°C for 30 min to inactivate complement. KIM6*^+^* wild type, the Δ*ail* mutant, and the Δ*ail*/*ail^+^* control strains were incubated in LB broth, with or without Kn or Cm, at 28°C with aeration until midlogarithmic phase (OD_600_ = 0.8). Cells were collected (4,000 × *g*, 5 min, 4°C), washed twice in PBS and diluted 100-fold. Bacterial suspensions were combined with an equal volume of NHS, normal BALB/c mouse sera (NMS), a 1:1 mix of NHS and MNS, or respective HIS, and incubated at 28°C. Viable bacteria were enumerated by plate count after 0, 60, or 150 min incubation. In some experiments, bacterial suspensions were pretreated with an equal volume of PBS or NMS for 1 h at 28°C. Next, 50% NHS or human HIS were added to bacteria and incubated for 90 min at the same temperature. Viable bacteria were enumerated by plate count at 0 or 90 min.

### Preparation of fleas.

O*ropsylla* montana female fleas were collected from growing colonies and prestarved for 4 d before the infection. Fleas were kept at 20–23°C and 75 −80% relative humidity.

### Preparation of infectious blood meal.

KIM6*^+^* wild type, the Δ*ail* mutant, the Δ*ail*/*ail^+^* control were incubated in BHI broth, with or without Kn or Cm, at 28°C overnight with aeration. Bacteria were collected and frozen in 10% glycerol at −80°C. Bacteria in the stock cultures were quantified by plate count on BHI agar. On the day of the experiment, bacteria were thawed, washed in PBS, and resuspended with defibrinated Swiss Webster mouse whole blood (Bioreclamation, Jericho, NY) to a final concentration of 1.0 − 2.0 × 10^9^ CFU mL^−^.

### Infection and maintenance flea feeds.

An adult mouse skin affixed to an artificial feeding device maintained at 37°C was used (23). In the infectious blood meal, fleas were allowed to feed for 1 h. To determine if Ail affected colonization insects fed blood containing the *Δail* mutant, Y. pestis KIM6^+^, or the *Δail*/*ail*^+^ complemented strain were collected, cold immobilized, and fleas that took a blood meal were identified under a dissecting microscope by the presence of fresh blood in the proventriculus or midgut. Three days after the infectious feed a 10 fleas/strain were collected and frozen at −80°C for later determination of bacterial numbers (designated 72 h). This experiment was repeated four times on separate days. Remaining insects were fed with sterile defibrinated Swiss Webster mouse whole blood (Bioreclamation) or defibrinated whole human blood (Bioreclamation) and selected as above. Fleas were kept for another 24 h in a maintenance chamber, collected, and frozen at −80°C (designated 96 h). To evaluate infection prevalence and infection rate, fleas were thawed, individually homogenized in 100 μL of a 10% glycerol solution in BHI, plated on BHI agar, and incubated 48 h at 28°C. Fleas were designated uninfected when no colonies grew from plating 15% of flea lysate. The latter experiment was repeated four times on separate days. All human blood used in these flea experiments was shown to have bactericidal activity against Y. pestis by using the serum resistance assay protocol described above.

### Sequence alignment and structural modeling.

Y. pestis and Y. pseudotuberculosis Ail sequences in GenBank (National Health Institute, NIH) as of October 2021 were analyzed with ClustalW for multiple sequence alignment. The deep learning-based modeling method, RoseTTAFold, was used to generate Ail structural models that were superimposed and compared with UCSF Chimera plug-in, MatchMaker (69, 70).

### Generation of pAil^F100V E108_S109insS^.

Y. pestis Pestoides F genomic DNA was used as a template to amplify the *ail* variant, *ail*_F100V E108_S109insS_. Primers complementary to the upstream (76 bp) and downstream regions (99 bp) were used: 5’GCAGGAGCTCTCATGTCAGATATTTG3’ (forward primer); 5’ATACGAGCTCTAGCCTACCCCTATTA3’ (reverse primer). The generated PCR product (750 bp) was cloned into pCR2.1-TOPO (Invitrogen, Carlsbad, CA).
